# Involvement of small G protein RhoB in the regulation of proliferation, adhesion and migration by dexamethasone in osteoblastic cells

**DOI:** 10.1371/journal.pone.0174273

**Published:** 2017-03-21

**Authors:** Fei Diao, Kangyao Chen, Yan Wang, Yidong Li, Weidong Xu, Jian Lu, Yu-Xia Chen

**Affiliations:** 1 Department of Pathophysiology, Second Military Medical University, Shanghai, China; 2 Department of Orthopedics, Changhai Hospital affiliated to Second Military Medical University, Shanghai, China; 3 Department of -Orthopedics, Fuzhou Second Hospital affiliated to Xiamen University, Fuzhou, Fujian Province, China; University of Texas Southwestern Medical Center, UNITED STATES

## Abstract

Long-term exposure to therapeutic doses of glucocorticoids (GCs) results in bone remodeling, which frequently causes osteoporosis and fracture healing retardation because of the abnormality of osteoblastic proliferation and differentiation. The mechanisms of GCs’ effect on osteoblasts are largely unknown. In this present study, we found that dexamethasone (Dex) could induce the expression of the small G protein, RhoB, in mRNA and protein levels in the osteoblast-derived osteosarcoma cell lines MG-63. The up-regulation of RhoB mRNA by Dex mainly occurs at posttranscriptional level by increasing its mRNA stability through PI-3K/Akt and p38 mitogen-activated protein kinase signaling pathways. Over-expression of RhoB in MG-63 cells magnified while down-regulation of RhoB level by RNA interference impaired Dex-induced growth inhibition but not differentiation. What’s more, over-expression of RhoB mimicked the effect of Dex on cell adhesion and migration. And interfering RhoB expression partially suppressed Dex-induced pro-adhesion and anti-migration in MG-63 cells. In conclusion, these results indicate that RhoB plays an important role in the pathological effect of Dex on osteoblastic growth and migration, which is a part of the mechanisms of GCs’ adverse effect on bone remodeling.

## Introduction

Glucocorticoids (GCs) regulate a wide variety of biological processes, including inflammation, immune response, cell proliferation, differentiation and apoptosis, thus are frequently used in the treatment of numerous diseases. The effects of GCs are mainly mediated by glucocorticoid receptor (GR), a ligand-dependent transcriptional factor that positively or negatively regulates the transcription of target genes by binding to the GC response elements (GREs) in the promoter or by interacting with other transcription factors such as p65 (NF-ĸB subunit) and AP-1[1–3]. In addition, GCs can also regulate gene expression through post-transcriptional mechanisms, such as the alteration of mRNA turnover or translation. These can be achieved in part through inhibition of signaling pathways related to serine/threonine kinase cascades, such as extracellular signal-regulated kinase (ERK), c-Jun N-terminal kinase (JNK), p38 and the IĸB kinases [4]. For example, GCs decrease mRNA stability of vascular endothelial growth factor (VEGF) gene in JNK dependent style in keratinocytes [5] and accelerate cyclooxygenase-2 (COX-2) mRNA decay through inhibiting p38 activation [6].

However, long-term clinical application of GCs is frequently limited by the metabolic side-effects. Continued systemic exposure of GCs causes not only osteoporosis, increased risk of fracture but also delayed fracture healing, a pathological process characterized by the decrease of bone remodeling [7–9]. The disability of bone formation is mainly attributed to the decrease of the cell number and the functions of osteoblasts, such as matrix synthesis and mineralization [10]. GCs have been shown to exert antiproliferative effect in most osteoblast cell contexts including MG-63 [11], G-292 [12] through activating GR. Therefore, the adverse effects of GCs on fracture healing may be due to the inhibition of osteoblast proliferation. However, the downstream effectors of GR-mediated action on osteoblast cells, are not fully understood.

Small GTPases of the Rho subfamily have been implicated in many physiological and pathological processes, including cell adhesion, motility, proliferation, survival and inflammation [13, 14]. In the subfamily, RhoB exhibits distinct expression patterns and biological functions compared to RhoA and RhoC. For example, both RhoA and RhoC are constantly expressed in the cells, while RhoB is an early response gene regulated by various stimuli including growth factors (i.g. TGFß, EGF), chemotherapeutic drugs (i.g. cisplatin and 5-FU), genotoxic stress, hypoxia, steroid and lipopolysaccharide [15–20]. RhoB functions as tumor suppressor in that loss of RhoB is frequently correlated with enhanced migration and invasion of cancer cells [14, 21, 22]. Our previous study has shown that RhoB is upregulated by Dex and is involved in Dex-induced anti-proliferation effect in human ovarian cancer cell lines [23]. Interestingly, in an attempt to identify the potential target genes responsible for glucocorticoid-induced osteoporosis, RhoB was speculated to be one of Dex-induced participants in mouse preosteoblast cell line MC3T3-E1 [24].

However, the functional role of RhoB in osteoblast biology and its contribution to GC-induced osteoblastic remodeling remain unclear. In this study, we demonstrate that RhoB expression is upregulated by Dex treatment in the osteoblastic cell line MG-63 through inhibition of it’s mRNA decay, which was related to the activation of Akt and p38 signals. Furthermore, the upregulation of RhoB mediates the effects of Dex on osteoblastic cell growth, migration and adhesion.

## Materials and methods

### Cell culture

Human osteosarcoma cell line MG-63 was obtained from China Infrastructure of Cell Line Resources (No. 3131C0001000700124), and cultured in MEM-EBSS (Life Technologies) supplemented with 10% heat-inactivated fetal calf serum (FCS). For detection of RhoB expression, cell proliferation, adhesion and migration, cells were grown to subconfluence in culture dishes for 24 h, then washed with PBS for twice followed culture in 5% charcoal-dextran stripped FCS with ethanol or different concentrations of Dex (Sigma-Aldrich Chemicals) for the indicated time.

### Western blotting

Western blotting was conducted as described [23]. Briefly, whole cell extract was prepared with lysis buffer (10 mM Tris, pH 7.5, 0.1 mM EDTA, 0.1 mM EGTA, 0.5% SDS, 0.1 mM ß-mercaptoethanol, containing 2 μg/ml of each of the protease inhibitors leupeptin, aprotinin, and pepstatin). We resolved lysates on 8~15% SDS-PAGE and immunoblotted the nitrocellulose membrane with the antibodies. The blot was then detected by chemiluminescence (ECL, Amersham Pharmacia Biotech. Arlington Heights, IL). Antibodies against RhoB, total-Akt, phospha-Akt were from Santa Cruz Biotechnology, ß-actin was from Sigma-Aldrich Chemicals, and antibodies against total-p38, JNK, ERK or phospho-p38, JNK and ERK were purchased from Cell signal Technology.

### Real-time PCR

Total RNA was extracted with the TRIzol reagent (Invitrogen) and 2 μg of total RNA was subjected to synthesize first-strand cDNA by Reverse Transcription System (Fermentas) in accordance with the manufacturer's instructions. Quantitative real-time PCR was performed with a real-time PCR detection system (Bio-Rad Laboratories, Hercules, USA) using SYBR Green I. The reaction volume was 10 μl and included a mixture of 20 ng cDNA, 2×PCR buffer mix and 0.1 μM each of 5’ and 3’ primers. The RhoB primers were 5’-TGCTGATCGTGTTCAGTAAG-3’ (sense) and 5’-AGCACATGAGAATGACGTCG-3’ (antisense). The amplifying reactions included 40 cycles at 94°C (30 sec), followed by 54°C (20 sec) and 72°C (30 sec), and a final extension for 10 min at 72°C. The relative-fold changes in RhoB mRNA expression were calculated according to a ΔΔCt method normalized against the internal control glyceraldehyde-3-phosphate dehydrogenase (GAPDH).

### Transfection of MG-63 cells

The mammalian expression plasmid of human RhoB (pcDNA3-RhoB) was generously provided by Dr. GC Prendergast. RNA interference construct for human RhoB (named RhoB-RNAi) was constructed as described previously [23]. Cells were cultured in 24-well plates (5×10^4^ cells) or 35-mm dishes (4×10^5^ cells) overnight and then transiently transfected with 2~4 μg of the blank vector (pcDNA3, RNAi-control), RhoB-wt, or RhoB-RNAi using LipofectAMINE Plus reagent (Invitrogen) according to the manufacturer’s instructions. 48 h after transfection, cells were treated with various concentrations of Dex for the indicated time for further experiments.

### Proliferation assay

Cell proliferation was measured by cell counting. Briefly, cells were seeded at 5×10^4^ cells per well in 24-well culture plates in triplicate, and cultured in DMEM containing 5% charcoal-dextran stripped FCS. Dex or ethanol vehicle was added and refreshed every other 24 h. After culture for indicated time, the cells were digested by 0.25% trypsin and counted with counting chamber after stopping with FCS.

### Alkaline phosphatase activity assay

Alkaline phosphatase (AKP) activity was determined as described [25]. Briefly, after treatment with Dex at 100 nM for 48 h, the cells were washed with PBS, trypsinized and homogenized in 0.25 M sucrose with a Teflon homogenizer. The homogenates were centrifuged at 3000 g for 5 min at 4°C. AKP activity in supernatants was determined by measuring the release of phenol from disodium phenyl phosphate spectrophotometrically (520 nm) at 37°C, expressed as the generation of phenol in nmol/min/mg protein. Total protein was measured with Lowry’s Method.

### Cell adhesion assay

Cell adhesive ability to ECM was determined by cell adhesion assay. Briefly, the cells were incubated in the medium containing the agents for the indicated time before reseeding into 96-well plates (1×10^4^ per well), which was precoated with 10 mg/ml fibronectin (Calbiochem, Darmstadt, Germany). After incubation at 37°C for another 1 h, the plates were gently washed twice with PBS to remove the unattached cells, and then the cell number was determined by MTT assay.

### Cell migration assay

Cell migration assay was performed using transwell chamber (8 μM pore size, Corning Incorporated, Corning, NY). MG-63 cells transfected with different plasmids expressing RhoB-wt, or RhoB-RNAi or their mock controls were treated with or without 100 nM Dex for 24 h. Then, 1×105 cells were resuspended in culture medium without serum, added into the upper compartment of the chamber. Culture medium with 10% FBS was added in the lower chamber, and the whole chamber set was then incubated at 37^°^C and 5% CO_2_ for 18 h. The insert set was removed and non-penetrating cells on the upper side of the compartment were gently wiped off with cotton swab. After fixation in 4% paraformaldehyde for 10 min, the insert was stained with 0.3% crystal violet for 5 min, and the cells on its lower side were counted under the microscopy.

### Statistics

The data are indicated as means ± S.D.. Statistical analysis was performed using the unpaired 2-tailed Student’s t-test or one-way analysis of variance with Dunnet’s post hoc test, and the significance level was set at p< 0.05.

## Results

### Dex increases the expression of RhoB through GR in osteoblasts

**Fig 1:**
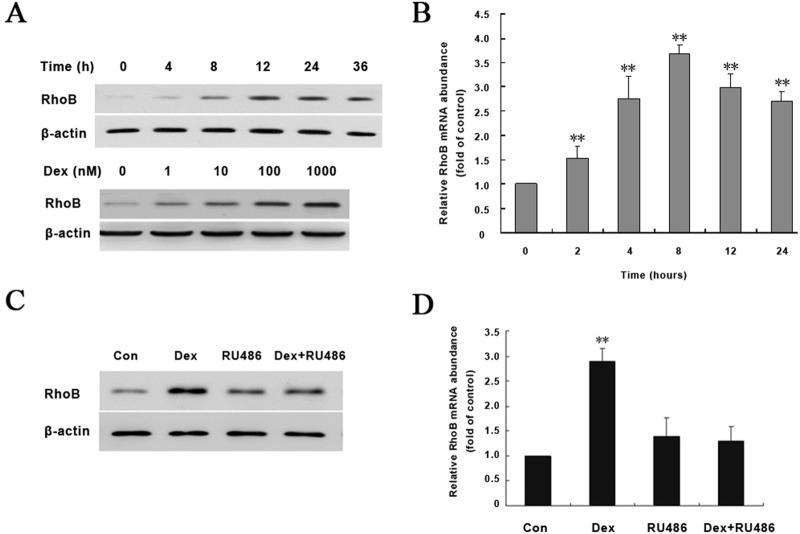
GR mediates Dex-induced RhoB expression in human osteoblast cells. MG-63 cells were cultured in medium containing 5% dextran-coated charcoal treated fetal calf serum with 100 nM Dex for different time intervals (0-36h), or with different doses of Dex (1–1000 nM) for 12 h. The control cells were treated with equal volume of ethanol. (A) Whole cell extract was used for detection of RhoB protein by Western blot with β-actin as a loading control. The blots represent one of three independent experiments. (B) Total RNA was isolated and aliquots cDNA were used to amply RhoB by real-time quantitative RT-PCR. The value represents means ± S.D. of increased folds against control level (0 h). (C and D) MG-63 cells were treated with either ethanol vehicle or 100 nM Dex in the absence or presence of 1 μM RU486 for 12 h for detection of RhoB protein (C) or mRNA (D). RU486 was added 1 h prior to addition of Dex. Symbol ** represents p<0.01 compared with control level (0 h).

To determine whether GC regulates RhoB expression in osteoblasts, we first use Dex to treat human osteoblast-like cell line MG-63, and detect RhoB protein level by Western blotting. It shows that following treatment of cells with 100 nM Dex, the expression of RhoB protein increased at 8 h, reached highest level at 12 hand maintained up to 36 h. Dex exhibited dose-dependently upregulting RhoB protein expression, with an effective dose as low as 1 nM and the maximal induction at 1000 nM (Fig 1A). As shown in Fig 1B, Real-time RT-PCR showed that RhoB mRNA level was rapidly up-regulated after 2h of 100 nM Dex treatment and reached the maximal level at 8 h (about 3.5-fold), then declined but still higher than control after 24 h of exposure.

To determine whether the up-regulation of RhoB expression by Dex was mediated by GR, MG-63 cells were pretreated for 1 h with 1μM RU486, a glucocorticoid receptor antagonist, and then treated in combination with 100 nM Dex for another 8 h (for mRNA detection) or 12 h (for protein detection). RU486 treatment alone did not affect expression of RhoB. However, it could dramatically block Dex-induced RhoB upregulation (Fig 1C and 1D), suggesting that the action of Dex on RhoB expression is mediated by GR.

### Akt and p38 pathways are required for Dex-induced RhoB expression

**Fig 2:**
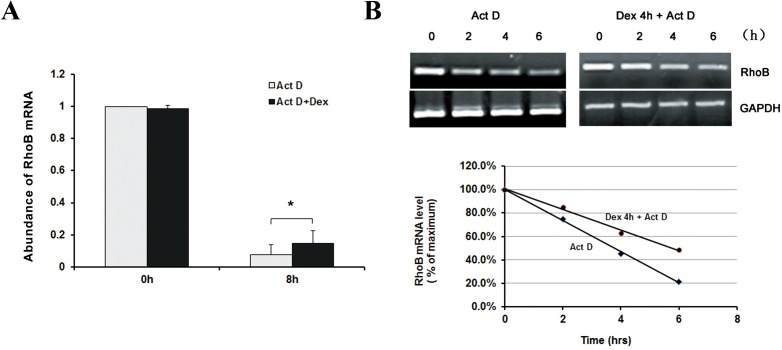
Up-regulation of RhoB expression by Dex occurs at posttranscriptional level. (A) MG-63 cells were pretreated with 5 μg/ml actinomycin D for 2h, and then treated with or without 100 nM Dex for 8 h. The RhoB mRNA abundant was detected by real-time quantitative RT-PCR with GAPDH as an internal control. (B) Blots and graph show effects of Dex on RhoB mRNA stability. MG-63 cells were pretreated by 100 nM Dex or equal volume of ethanol for 4 h, and then treated with 5 μg/ml actinomyc in D for 0, 2, 4 and 6 h. Decay of RhoB mRNA abundance was detected by both semi-quantitative and real-time quantitative RT-PCR. Results for RhoB mRNA are shown in a regression curve. Data are means of three separate experiments.

To examine whether the induction of RhoB by Dex occurred at transcriptional or post-transcriptional level, MG-63 cells were pre-treated with actinomycin D for 2 h to stop RNA synthesis prior to the exposure to 100 nM Dex for 8 h. It showed that RhoB mRNA level decreased with the time in both of the control cells and Dex-treated cells, and the abundance in Dex treated cells was still about 1.9-fold of that of control cells (Fig 2A). These indicate that the effect of Dex on RhoB mRNA expression may mainly due to increased mRNA stability. Therefore, RhoB mRNA half-life was detected to verify this hypothesis. Cells were pretreated with 100 nM Dex for 4 h, and followed by 5 μg/ml actinomycin D for further 6 h. RhoB mRNA abundance was determined every two hours. As we expected, RhoB mRNA abundance dropped to 50% at ~3 h following actinomycin D administration in control cells. While in Dex pre-treated cells, RhoB mRNA half-life prolonged to ~5 h (Fig 2B). It suggests that Dex induction of RhoB mRNA may mainly due to its decreased degradation rate.

**Fig 3:**
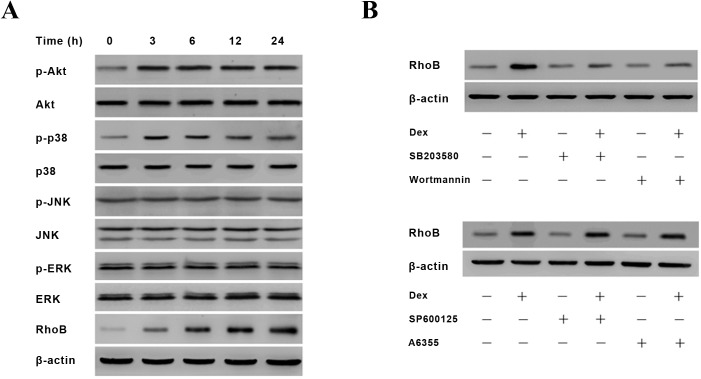
PI-3K/Akt and p38 signaling pathways are involved in RhoB expression by Dex. (A) MG-63 cells were maintained in DMEM medium containing 5% dextran-coated charcoal treated fetal calf serum for 24 h and then treated with 100 nM Dex for different time intervals, cells were then harvested for detection of RhoB, phosphorylated or total Akt, p38, JNK, and ERK by Western blot. (B) MG-63 cells were pre-incubated with or without 15 μM SB203580 or 10 μM Wortmannin or 15 μM SP600125 or 15 μM A6355 for 1h and then treated with 100 nM Dex for 12 h. Cells were then harvested for detection of RhoB protein by Western blot. β-actin was detected for loading control. The blots represent one of three independent experiments.

To determine the signal pathways participating in Dex/GR-induced RhoB expression, we further examined the phosphorylation status of Akt, p38, JNK and ERK in Dex-treated MG-63 cells by Western blot. It can be seen in Fig 3A that, following 100 nM Dex treatment, the phosphorylated Akt and p38 increased significantly. However, the levels of p-JNK and p-ERK did not change after Dex treatment. Furthermore, treatment of cells with Akt inhibitor (Wortmannin) or p38 inhibitor (SB203580) obviously suppressed Dex’s induction of RhoB protein, while JNK inhibitor (SP600125) and ERK inhibitor (A6355) did not affect Dex’s effect on RhoB expression (Fig 3B). It suggests that up-regulation of RhoB expression by Dex is Akt and p38 signal pathways dependant.

### Involvement of RhoB in the inhibition of MG-63 proliferation by Dex

**Fig 4:**
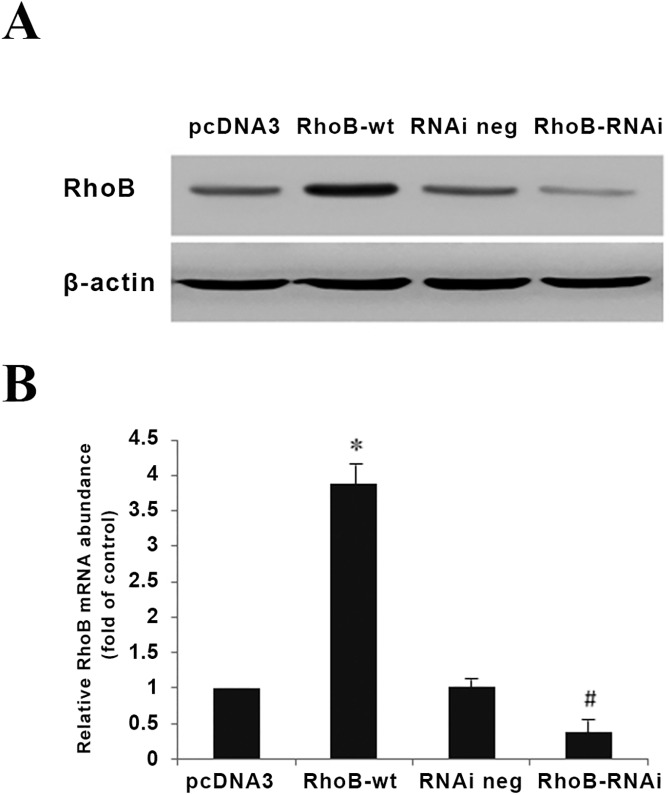
Characterization of RhoB expression in transfected MG-63 cells. (A) MG-63 cells were transiently transfected with different expression plasmids (pcDNA3, RhoB-wt, RNAi-neg, or RhoB-RNAi). 48 h later, the expression of RhoB protein was examined by Western blot as described in “Materials and methods”. The results are representative of three independent experiments. (B) Densitometry analysis of the Western blot. Data are means ± S.D. of three independent experiments. Symbol * represents p<0.05 compared with pcDNA3 group. Symbol ^#^ represents p<0.05 compared with RNAi-neg group.

To further understand the bioactivity of Dex-induced RhoB in osteoblastic cells, cell systems with RhoB protein over-expression or down-expression were conducted by transiently transfecting MG-63 cells with RhoB construct (RhoB-wt) or RhoB interfering RNA construct (RhoB-RNAi) (Fig 4).

**Fig 5:**
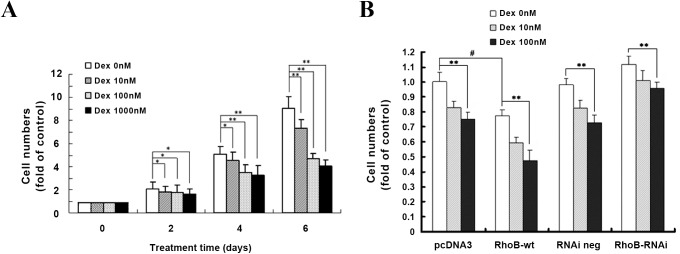
RhoB signaling is involved in the proliferation inhibition of Dex in MG-63 cells. (A) MG-63 cells were cultured in medium containing 0–1000 nM Dex for 0, 2, 4 and 6 days, respectively. (B) The transfected cells were cultured in medium containing 5% dextran-coated charcoal treated fetal calf serum for 24 h, then changed to the same medium supplied with 0–100 nM Dex and cultured for 4 days. The proliferation in monolayer culture was monitored by cell number counting. Data are means ± S.D. of four independent experiments. Symbol * or ^#^represents p<0.05, ** represents p<0.01.

We confirmed firstly in our experimental system that, Dex treatment for above 2 days could significantly suppress the growth of MG-63 cells in a dose dependent manner (Fig 5A). Cell proliferation was then investigated in RhoB-wt and RhoB-RNAi cells. It shows that over-expression of RhoB resulted in a ~16.9±2.3% inhibition of cell number in comparison to that of the blank vector cells. This result indicates that RhoB itself could play a negative role in regulating MG-63 cell growth, supporting our supposition that the up-regulation of RhoB mediated the growth inhibition by Dex. However, there is no significant difference in the cell proliferation between RhoB-RNAi cells and control cells, excluding a physiological role of endogenous RhoBinMG-63 cell proliferation. Based on the fact that Dex increases the level of RhoB mainly by prolonging its half-life, we then further investigated whether cultured the transfected cells with Dex could aggravate growth inhibition. The result showed a more notable cell proliferative inhibition rate 35.6±1.9% at 100 nM Dex) when RhoB-wt cells were treated with Dex for 4 days as compared with that of the blank vector cells 25.1±2.1% at 100 nM Dex). And treatment of RhoB-RNAi cells with 100 nM Dex still suppressed cell growth by 17.9±1.3% (Fig 5B). These results indicate that RhoB is involved in the growth inhibition of Dex in MG-63 cells.

Using a widely accepted osteoblastic differentiation marker-alkaline phosphatase (AKP), we then examined whether RhoB participates in differentiation regulated by Dex in MG-63 cells. The activity of AKP was elevated by Dex in the blank vector transfected cells by 2.89-fold compared to the ethanol-treated control, while Dex showed no more induction in AKP activity in RhoB-wt transfected cells (data not shown). These results suggest that RhoB may have no effect on Dex-induced osteoblast cell differentiation.

### RhoB contributes to Dex-regulated cell adhesion and migration

**Fig 6:**
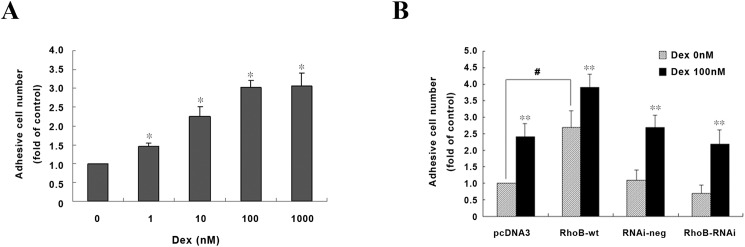
RhoB participates in pro-adhesion by Dex in MG-63 cells. Cells were pretreated for 48 h with 0–1000 nM Dex (A) or with 100 nM Dex (B) before seeded onto 96-well dishes pre-coated with 10 μg/ml fibronectin. 60 min later the cells were washed three times with PBS, and the remaining attached cell number were determined. Data are means ± S.D. of three independent experiments. Symbol ^#^ represents p<0.05, ** represents p<0.01 vs. Dex 0 nM in each group.

Considering that Rho GTPase protein has a remarkable role in regulating cellular skeleton, which may affect cell adhesive and migratory ability, we next assessed the potential effect of RhoB on those cellular behaviors. As shown in Fig 6A, Dex treatment at low concentration (1 nM) resulted in a significant increase in MG-63 cell adherence to fibronectin and the stimulatory effect was about 3-fold at 100 nM as compared to the control. Interestingly, RhoB overexpression alone led to a 2.53-fold increase in MG-63 cell adhesion to fibronectin compared with control in the absence of Dex (Fig 6B), and presence of Dex further increased the attached cells by 61±3.4% compared with the RhoB-wt cells without Dex treatment. RhoB knock down correspondently reduced adhesive cell number by 21±2.9% when compared to the control, however, Dex still induced cell adhesive ability by 2.1-fold in RhoB-RNAi cells.

**Fig 7:**
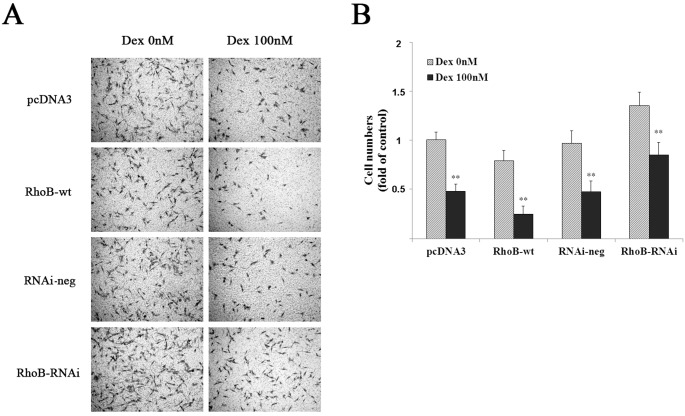
RhoB participates in the migration inhibition by Dex in MG-63 cells. (A) The transfected cells were pretreated with or without 100 nM Dex for 24 h, and then resuspended for cell migration assay as described in “Materials and methods”. (B) Data shown are the average cell number counted from 5 visual fields for each corresponding cells. Data are means ± S.D. of three independent experiments. Symbol ** represents p<0.01 vs. Dex 0 nM in each group.

Using transwell chamber, we found that in the both mock transfected cells, the number of cell migrating to the lower side of the membrane in 100 nM Dex-treated group is about 49±2.9% less than that of ethanol-treated control. Over-expression of RhoB mimicked to some extent the inhibition effect of Dex on MG-63 cell migration, and showed more notable inhibition effect (58±3.2% inhibition rate) in the presence of Dex. And down-regulation of RhoB protein by transfecting RhoB-RNAi could impair partially the migration-inhibition ability of Dex, although Dex still suppressed significantly the migrated cell number by 38±4.3% (Fig 7). These results indicate that RhoB partially mediates Dex’s effect on MG-63 cell adhesion and migration.

## Discussion

It has been well established that the reduction of functional osteoblasts is one of the causative factors of GC-caused osteoporosis and fracture healing delay. This reduction is caused by a combination of multiple factors, which include GC-induced apoptosis, trans-differentiation into adipocytes, and impairment in the mitogenic response of preosteoblasts to growth factors [26–29]. Several labs have observed the growth inhibition effects of Dex on osteoblast cells including MG-63 cells, although apoptotic event was not affected [11, 30]. The possible mechanisms for the growth suppression by Dex may involve the activation of glycogen synthase kinase-3β; the upregulation of MAPK phosphatase (MKP-1), which leads to dephosphorylation of ERK and the inactivation of cell cycle machinery [11, 26, 31]. Our data showed that up-regulation of RhoB is involved in Dex-induced cell growth in MG-63 cells, which provides another possible mechanism of the anti-proliferation action of Dex in these cells. Since previous study showed that GC induces the differentiation of osteoblast [32–35], and MG-63 is widely used for the study of osteoblastic differentiation, we therefore determined the role of RhoB in the differentiation effect of Dex. Using AKP as a differentiation marker, we showed that Dex induced AKP activity as expected, but change of RhoB level has no apparent effect on both basal and Dex-induced AKP activity, indicating that Dex-induced osteoblast cell differentiation is independent of RhoB.

Delay of fracture repair is another common side-effect of long-term GC therapy, evidenced at least in a rabbit ulnar osteotomy model [36] and rat closed femur fracture model [37]. The osteoblast cells play a key role in the fracture healing process. While the fracture occurs, the osteoblast cells nearby migrate to the fracture location, multiply and synthesize the extracellular matrix, induce mineralization, thus promote the formation of hard callus [8, 27]. Although the effect of GC on bone metabolism is well established, the underlying mechanisms for GC-induced delay of fracture healing are rarely clarified. We demonstrated that Dex treatment could significantly result in an increase in MG-63 cell adhesion to ECM and a decrease in the cell motility besides to cell growth inhibition. These effects may explain the negative effect of Dex on fracture healing process. Overexpression of RhoB boosted while decrease of RhoB expression reversed to some extent such effects of Dex in the cells, suggesting that RhoB signal pathway at least mediates partially the regulation of osteoblast cell adhesion and motility by Dex.

In the present study, we demonstrated that Dex treatment resulted in a remarkable increase in RhoB expression in human osteoblastic cell line and the effect is mediated by GR. It is well known that GCs regulate the expression of genes containing GREs in the promoter region by recruiting GR to the binding elements. Notably, our experiment demonstrated Dex could notably prolong the half-life period of RhoB mRNA, Moreover, the activity of a luciferase reporter gene containing a ∼1.9 kb (−1756/+111) fragment of the promoter sequence of human RhoB gene was not induced by Dex (data not shown), suggesting that unlike TGFß or genotoxic stresses which regulates RhoB expression by recruiting downstream effectors such as smad3 or NF-Y to the CCAAT element in RhoB promoter [18, 38], the modulation of RhoB mRNA expression by Dex may mainly occur at posttranscriptional level. However, the transcription inhibitor—actinomycin D couldn’t block totally the upregulation of RhoB mRNA expression by Dex, indicating that the possibility of transcriptional regulation still exists. Using Affymetrix oligonucleotide array, Chen et al. declared a putative GRE (ACAATATGTAC) in the promoter region (-3721 to -3711 bp) of murine RhoB gene, which may be responsible for Dex-induced murine RhoB mRNA expression [39]. Although there have been no direct data so far that identified this half site of GRE as essential nucleotides for GC-induced RhoB transcript, the possibility that Dex mediates RhoB mRNA expression via its modulation in transcriptional level still can’t be excluded.

Recent works have demonstrated that GC can regulate the activity and /or expression of particular kinases and phosphatases, thus affecting the signaling efficacy toward the modulation of gene expression and their mRNA stability. For example, depending on the cell type used, GC has been shown to activate or suppress p38, JNK, ERK or PI-3K/Akt pathway [40–44]. The p38 pathway has been particularly linked to the promotion of increased mRNA stabilization of numerous inflammatory genes, such as IL-1, IL-6, IL-8, VEGF, TNFα, and COX-2 [4]. Our previous study showed that p38 pathway is involved in heat stress-induced RhoB expression in human lung carcinoma cell line A549 [19]. In the present study, we found that Dex can activate Akt and p38 in MG-63 cells. Akt and p38 -specific inhibitors—Wortmannin or SB203580 significantly inhibited Dex-induced RhoB protein expression, indicating that the PI-3K/Akt and p38 pathways are involved in Dex-induced RhoB mRNA stabilization. However, in Hela cells, GC accelerated COX-2 mRNA decay was demonstrated as a result of directly inhibition of p38 phosphorylation, which in turn responsible for the decrease of COX-2 mRNA stabilization [6]. In addition, we didn’t detect the regulatory effect of Dex on the ERK phospharylation in the presence of serum. Interestingly, it is reported that Dex attenuated serum-stimulated ERK activation in MG-63 cell [11] but had no effect on ERK activity in human airway smooth muscle cells [45]. The somewhat contradictory data indicate that the regulatory events of GC on these kinase pathways are in a cell type-specific fashion, and to some extent, are affected by the in vitro experimental condition.

In conclusion, our results demonstrate that Dex increases the small G protein RhoB expression mainly by increasing its mRNA stability through Akt and p38 signal pathways in the osteoblast cells MG-63, which partially mediates Dex-induced inhibition of cell growth, adhesion to ECM and suppression of cell migration. All of these phenomena may account for the side-effects of GC on the bone metabolism, such as delay of fracture healing. However, further experiments using bone-derived primary cultures and animal models to mimic the clinical settings are needed to verify our proposal.
